# Dexmedetomidine preconditioning attenuates ferroptosis in myocardial ischemia-reperfusion injury via α2 adrenergic receptor activation

**DOI:** 10.1016/j.heliyon.2024.e39697

**Published:** 2024-10-22

**Authors:** Mingling Wang, Zhuoyu Ren, Xiaotong Sun, Yaozu Li, Zuolei Chen

**Affiliations:** a Department of Anesthesiology, Qingdao Women and Children's Hospital, School of Medicine, Shandong University, Jinan, China; b Shandong Provincial Medicine and Health Key Laboratory of Clinical Anesthesia, School of Anesthesiology, Shandong Second Medical University, Weifang, China; c Department of Anesthesiology, Pain and Perioperative Medicine, The First Affiliated Hospital of Zhengzhou University, Zhengzhou, China; d Department of Anesthesiology of the Affiliated Hospital of Qingdao Binhai University, Qingdao, China

**Keywords:** Dexmedetomidine, Ferroptosis, Myocardial ischemia-reperfusion injury, α2 adrenergic receptor, SLC7A11

## Abstract

**Objective:**

Dexmedetomidine (Dex) is a potent agonist of the α2 adrenergic receptor that has been shown to possess sedative and hypnotic properties. Dex can protect against myocardial ischemia-reperfusion injury (MIRI) by inhibiting ferroptosis. However, these studies were based on Dex post-conditioning, and the role of α2 adrenergic receptors in this process is unclear. In this study, we investigated whether Dex preconditioning can prevent MIRI by attenuating ferroptosis and whether this effect depends on α2 adrenergic receptors in rats.

**Methods:**

Male Sprague–Dawley rats were randomly assigned to five groups: sham (saline-treated), I/R (ischemia-exposed), Dex + I/R (Dex pre-treatment), Dex + Yoh + I/R (Dex and yohimbine pre-treatment), and Yoh + I/R (yohimbine pre-treatment). Cardiac function, myocardial infarction, and morphological changes were assessed. Transmission electron microscopy was used to analyze mitochondrial morphology. Ferroptosis-related indicators and lipid peroxidation were measured using western blotting and qRT-PCR.

**Results:**

Our findings indicated that Dex preconditioning improved cardiac function, reduced infarct size and apoptosis, and inhibited ferroptosis in the rat myocardium after MIRI. These effects were associated with the upregulation of Nrf2, SLC7A11, and GPX4 expression, as well as the downregulation of Ferritin, TFR1, ACSL4, COX2, IL-1β, IL-6, and TNF-α expression. Importantly, yohimbine, an α2 adrenergic receptor antagonist, abolished these protective effects.

**Conclusion:**

These results suggest that Dex preconditioning can prevent MIRI by attenuating ferroptosis via α2 adrenergic receptor activation and by modulating the Nrf2/SLC7A11/GPX4 pathway.

## 1 Introduction

Acute myocardial infarction (AMI) is a leading global health burden that causes significant mortality and morbidity [1]. Currently, timely and complete reperfusion is the most effective approach for improving the prognosis of patients with AMI [2,3]. However, reperfusion can cause paradoxical cardiomyocyte dysfunction, including reduced efficacy of myocardial reperfusion, increased infarction size, and myocardial ischemia-reperfusion injury (MIRI) [4]. Therefore, prevention and treatment of MIRI have important clinical implications for patients with myocardial infarction.

The pathogenesis of MIRI is multifaceted and involves various mechanisms, including calcium overload, reactive oxygen species (ROS) overproduction, inflammation, and endothelial dysfunction [5]. In addition, iron-mediated ferroptosis is believed to contribute to MIRI, and targeting ferroptosis may be a promising approach to prevent and treat MIRI [6,7]. Ferroptosis is an identified mode of cell death triggered by iron-dependent lipid peroxidation and was initially described by Dixon in 2012 [8]. Ferroptosis is primarily characterized by disruptions in iron metabolism, imbalances in the amino acid antioxidant system, and the accumulation of lipid peroxides [8]. It can contribute to MIRI by inducing lipid peroxidation, leading to oxidative injury and subsequent damage to organelles, including the mitochondria. Therefore, the inhibition of ferroptosis could provide substantial protection against MIRI.

Dexmedetomidine (DEX) is a selective agonist of α2 adrenergic receptors that produces sedative and hypnotic effects, making it a commonly used drug in perioperative and intensive care settings [9]. Dex reduces catecholamine release and the stress response, thereby protecting against ischemic and hypoxic injury, such as neuroprotection and renoprotection [10,11]. Dex also protects against MIRI and improves cardiac outcomes during non-cardiac surgery [[12], [13], [14]]. Yu et al. [15] reported that Dex alleviates DOX-induced cardiac dysfunction by inhibiting myocardial superoxide anions and mitochondrial ROS production. Dex also exhibited dose-dependent inhibition of iron overload, pro-inflammatory cytokines, and apoptosis in human neuroblastoma cells [16].

Interestingly, Wang et al. demonstrated that Dex attenuates MIRI-induced ferroptosis via AMPK/GSK-3β/Nrf2 or SLC7A11/glutathione peroxidase 4 (GPX4) activation [17,18]. However, these protective effects were observed after the post-conditioning treatment with Dex. It remains unclear whether Dex preconditioning can also prevent MIRI by inhibiting ferroptosis and whether this effect involves α2 adrenergic receptors. Thus, our study aimed to investigate the potential of Dex preconditioning to prevent MIRI by attenuating ferroptosis and to determine whether α2 adrenergic receptors are involved in this effect in rats. By addressing these questions, we aimed to clarify the mechanisms underlying the protective effects of Dex against MIRI and to identify potential targets for therapeutic interventions.

## 2 Materials and methods

### 2.1 Experimental animals

Male Sprague–Dawley rats weighing 220–240 g were procured from Jinan Pengyue Laboratory Animal Co., Ltd. (Jinan, China) and kept under controlled standard conditions. The experimental protocols were approved by the Committee on the Ethics of Animal Experiments at Weifang Medical University (2019SDL073). The rats were divided randomly into five groups: sham group (saline-treated sham-operated rats, i.p., 30 min before surgery), I/R group (ischemia-exposed rats treated with saline, i.p., 30 min before ischemia), Dex + I/R group (ischemia-exposed rats treated with Dex, 5μg/kg, i.p., 30 min before ischemia), Dex + Yoh + I/R group (ischemia-exposed rats treated with yohimbine (Yoh), an alpha 2-adrenergic antagonist, 500μg/kg, i.p., 35 min before ischemia, and Dex 5μg/kg, i.p., 30 min before ischemia), and Yoh + I/R group (ischemia-exposed rats treated with Yoh, 500μg/kg, i.p., 35 min before ischemia). The I/R model and the chosen dose were established according to a previously described method [19,20]. The left anterior descending coronary artery was occluded for 30 min (ischemia), followed by a subsequent release for 120 min (reperfusion). The sham group underwent an identical procedure, excluding coronary artery ligation.

### 2.2 Cardiac function evaluation, infarct size assessment and morphological observation

After 120 min of reperfusion, cardiac function was measured using an imaging system (Vevo 3100, VisualSonics). The area of myocardial infarction was determined using triphenyl tetrazolium chloride staining. The infarcted area (IA; white) and non-infarcted area (NIA; red) of the left ventricle were quantified using Image J software. Infarct size (IS) was calculated as IS = IA/(IA + NIA)%. Additionally, partial hearts were fixed in 4 % paraformaldehyde, embedded, and stained with hematoxylin and eosin. The resulting sections were examined and imaged under a microscope (BX-53; Olympus Corporation, Tokyo, Japan) to visualize and evaluate infarcted and non-infarcted areas of the ventricle.

### 2.3 Transmission electron microscopy

Transmission electron microscopy (TEM) was used to analyze the mitochondrial morphology. The heart was placed on ice immediately after removal. The affected tissue was then sliced along the long axis of the myocardial fibers and trimmed into cords (2 mm long, 1 mm^2^ cross-section), which were subsequently placed in 2.5 % glutaraldehyde. The tissues were fixed, dehydrated, embedded, sectioned, stained, and viewed under a transmission electron microscope (HT-7800). Ten micrographs were randomly captured for each rat.

### 2.4 Ferroptosis-related indicators and lipid peroxidation evaluation

The blood was drawn via cardiac puncture, allowed to clot at room temperature, and centrifuged at 3000 rpm for 10 min. The serum was aliquoted and stored at −80 °C until further analysis. Levels of malondialdehyde (MDA, Cat.E-BC-K025-M), glutathione (GSH, Cat. E-BC-K030-M), and glutathione disulfide (GSSG, Cat. E-BC-K097-M) were determined using ElabScience quantification kits (Wuhan, China). The total iron content and SOD activity were measured using an iron assay kit (Cat. E-BC-K772-M) and a T-SOD assay kit (Cat. E-BC-K020-M, Elabscience). Additionally, serum creatine kinase-MB (CK-MB), a biochemical marker of cardiomyocyte damage, was examined using an ELISA kit (Cat. H-197-1-2, Jiancheng Institute, Nanjing, China).

### 2.5 Western blot analysis

Protein extraction from the myocardial tissue was performed using RIPA buffer. Proteins were separated by 10 % sodium dodecyl sulfate-polyacrylamide gel electrophoresis and transferred onto a PVDF membrane. The membrane was blocked with 5 % skimmed milk powder for 2 h at room temperature.

Primary antibodies against COX2 (Cat. ab179800), transferrin receptor 1 (TFR1, Cat. ab269513), Ferritin (Cat. ab75973), ASCL4 (Cat. ab155282), SLC7A11 (Cat. ab175186), GPX4 (Cat. ab125066) (all from Abcam), and Nrf2 (Cat. 16396-1-AP, Proteintech) were incubated overnight at 4 °C. Subsequently, HRP-conjugated rabbit or rat secondary antibodies (1:2000; Abcam) were used for 1.5 h at room temperature. Signal detection was performed using ECL (CWBIO), and band intensities were analyzed using the ImageJ software. Rabbit anti-GAPDH antibody (Cat. 60004-1-Ig, Proteintech, 1:4000) was used as the loading control.

### 2.6 qRT-PCR

Myocardial tissue was processed for RNA extraction using TRIzol reagent. After extraction, chloroform was added, and the mixture was shaken and centrifuged. The aqueous phase was transferred to a new tube, and 75 % ethanol was added to precipitate the RNA. The sample was centrifuged again, the supernatant discarded, and the RNA pellet was briefly air-dried before being dissolved in RNase-free water. The quality and concentration of the RNA were measured using spectrophotometry (ND-2000c, NanoDrop). cDNA was synthesized from RNA using HiScript® III All-in-one RT SuperMix (Vazyme, Nanjing, China). For quantitative PCR, the ChamQ Universal SYBR qPCR Master Mix kit (Vazyme) was used with a real-time qPCR instrument (ABI7500, ABI, Foster City, CA, USA). The qPCR primers that were used are listed in Table 1.

**Table 1:** The primers used in the study.

Gene	Primer sequence (5’→3′)	
*SLC7A11*	F: TGCTGCCTACACAAAGACGTT	R: CGCCTTGCCCTTTAAGTATTCACC
*IL-6*	F: AATCTGCTCTGGTCTTCTGGAG	R: GTTGGATGGTCTTGGTCCTTAG
*IL-1β*	F: ACTATGGCAACTGTCCCTGAAC	R: GTGCTTGGGTCCTCATCCTG
*TNF-α*	F: GTCCCAACAAGGAGGAGAAGT	R: CTGGTATGAAATGGCAAATCG
*GAPDH*	F: GATGCTGGTGCTGAGTATGTCG	R: TGGTGCAGGATGCATTGCTGA

### 2.7 Statistical analysis

Data are presented as the mean ± SEM. The results were analyzed using GraphPad Prism 9.0. One-way analysis of variance followed by post hoc tests was used to compare multiple groups. Statistical significance was defined as *P* < 0.05.

## 3 Results

### 3.1 Dex preconditioning diminished myocardial infarct size and improved cardiac function

**Fig. 1:**
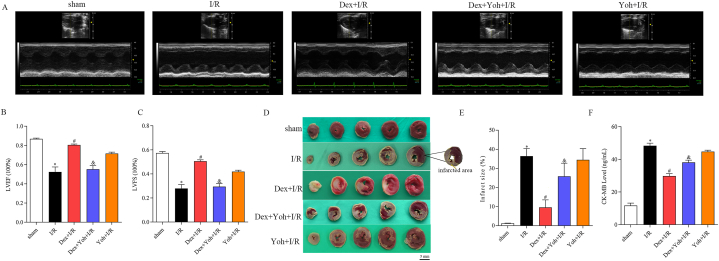
Dex preconditioning reduced myocardial infarct size and improved cardiac function. (A) Cardiac function was measured with echocardiography. (B) Left ventricular ejection fraction (LVEF). (C) Left ventricular fractional shortening (LVFS). (D) Myocardial infarction was detected by TTC staining. (E) Infarct size ratio. (F) CK-MB level examined by ELISA. n = 3–6/group. All data are presented as the mean ± SEM. ∗*P* < 0.05 *vs.* the Sham group. ^#^*P* < 0.05 *vs.* the I/R group. ^&^*P* < 0.05 *vs.* the Dex + I/R group.

Cardiac function was evaluated using echocardiography in the different groups of rats after 120 min of reperfusion. The I/R group showed impaired left ventricular wall motion and contractility and significantly decreased left ventricular ejection fraction (LVEF) and fractional shortening (LVFS) compared to the sham group. Dex pretreatment significantly increased LVEF and LVFS and improved ventricular wall motion and myocardial contractility. However, Yoh abolished the protective effect of Dex on cardiac function, with significant reductions in LVEF and LVFS (Fig. 1A–C). Moreover, 120 min after reperfusion, the I/R group had a significantly larger myocardial infarct size than that of the sham group. Dex preconditioning diminished the myocardial infarct size, whereas the Yoh + I/R group rats had a myocardial infarct size similar to that of the I/R group. The beneficial effects of Dex were lost when Dex was co-administered with Yoh (Fig. 1D and E). Consistent with this, serum CK-MB levels were significantly elevated in the I/R group, but Dex pretreatment attenuated the increase caused by I/R injury. However, when Dex was combined with Yoh, the serum CK-MB levels were restored. No significant difference in serum CK-MB levels was observed between the YOH + I/R and I/R groups (Fig. 1F).

### 3.2 Dex preconditioning improved morphology and myocardial ultrastructure

**Fig. 2:**
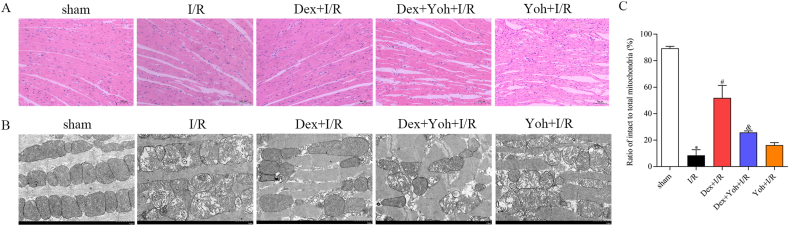
Dex preconditioning improved the morphology and myocardial ultrastructure. (A) Cardiac pathological injury was detected by HE staining ( × 100). (B) Ultrastructural changes of the myocardium and mitochondria were observed using transmission electron microscopy. (C) Ratio of intact to total mitochondria (%). n = 3/group. ∗*P* < 0.05 *vs.* the Sham group. ^#^*P* < 0.05 *vs.* the I/R group. ^&^*P* < 0.05 *vs.* the Dex + I/R group.

The sham group displayed normal morphology and arrangement of cardiomyocytes, with no evidence of edema, degeneration, or necrosis. Myofibers remained intact, and no signs of irregularities were detected. However, in the I/R group, cardiomyocytes exhibited a range of pathological features, including irregular morphology and arrangement, loss of transverse striations, edema, and neutrophil infiltration. In contrast, Dex pretreatment significantly attenuated these pathological changes. However, the Dex + Yoh + I/R and Yoh + I/R groups showed similar levels of myocardial damage as the I/R group (Fig. 2A).

Transmission electron microscopy demonstrated the effect of Dex preconditioning on ultrastructural changes in the myocardium and mitochondria. In the sham group, the myocardial structure was normal, with orderly arranged myocardial fibers and mitochondria with intact cristae. In contrast, the I/R group displayed a disordered and fragmented arrangement of myocardial fibers, accompanied by abnormal and disorganized mitochondria. Furthermore, reduced or absent cristae and vacuolization were observed in this group. Dex preconditioning preserved the structure of cardiomyocytes and mitochondria, with mild lysis of myocardial fibers and less disruption of the mitochondrial cristae. Similar to the I/R group, the Yoh + I/R and DEX + Yoh + I/R groups exhibited severe ultrastructural alterations in the myocardium (Fig. 2B and C).

### 3.3 Dex preconditioning altered the ferroptosis-related indicators

**Fig. 3:**
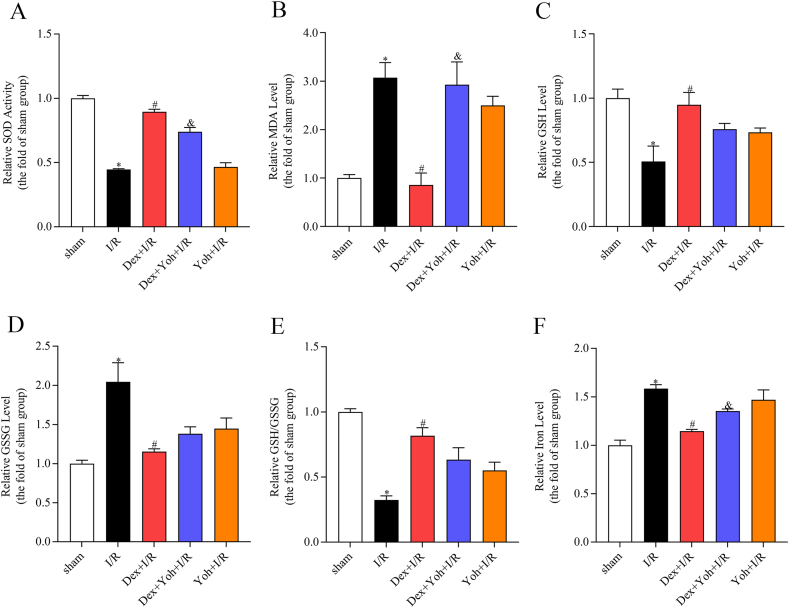
Dex preconditioning altered ferroptosis-related indicators. (A) SOD activity. (B) MDA level. (C) GSH level. (D) GSSG level. (E) The ratio of GSH to GSSG. (F) Iron level. n = 3/group. All data are presented as the mean ± SEM. ∗*P* < 0.05 *vs.* the Sham group. ^#^*P* < 0.05 *vs.* the I/R group. ^&^*P* < 0.05 *vs.* the Dex + I/R group.

The I/R group exhibited significantly elevated levels of MDA, GSSG, and iron, as well as reduced levels of SOD and GSH compared to the sham group. These changes were reversed by pre-treatment with Dex. The Dex + Yoh + I/R group had significantly higher MDA and lower SOD levels than the Dex + I/R group. The Yoh + I/R group did not show significant differences in serum MDA, SOD, GSH, and iron levels compared to the Dex + Yoh + I/R group (Fig. 3A–F).

### 3.4 Dex preconditioning mitigated MIRI by modulating ferroptosis-related proteins

**Fig. 4:**
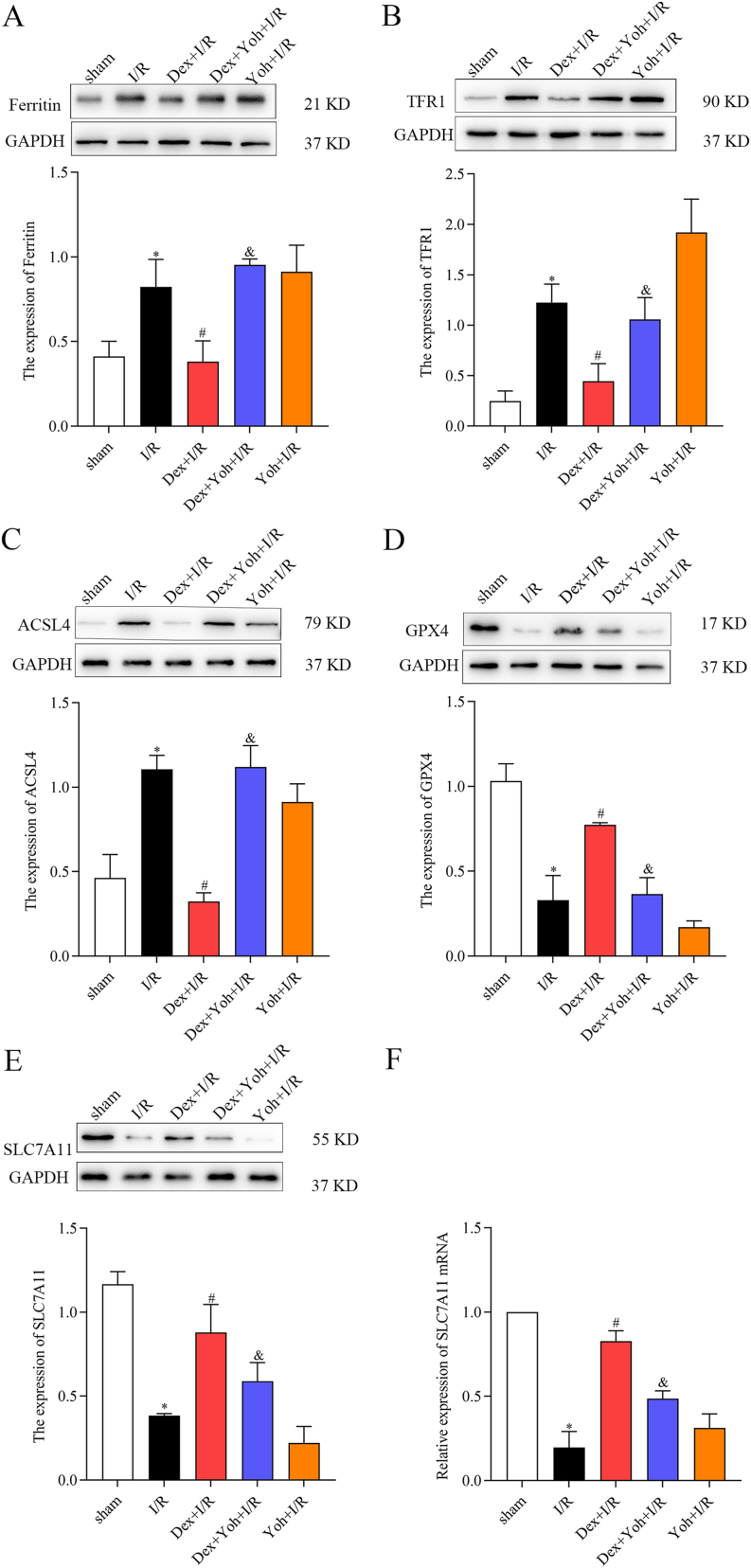
Dex preconditioning mitigated MIRI by modulating ferroptosis-related proteins and mRNA. (A) Ferritin protein level. (B) TFR1 protein level. (C) ACSL4 protein level. (D) GPX4 protein level. (E) SLC7A11 protein level. (F) SLC7A11 mRNA expression. n = 3/group. All data are presented as the mean ± SEM. ∗*P* < 0.05 *vs.* the Sham group. ^#^*P* < 0.05 *vs.* the I/R group. ^&^*P* < 0.05 *vs.* the Dex + I/R group.

In the I/R group, myocardial tissue exhibited upregulated expression of ferritin, TFR1, and ASCL4, along with downregulated expression of SLC7A11 and GPX4 compared to the sham group. Dex preconditioning reversed these changes, decreasing ferritin, TFR1, and ASCL4 expression and increasing SLC7A11 and GPX4 expression. Furthermore, in the Dex + Yoh + I/R group, there was an increase in ferritin and ASCL4 expression and a decrease in SLC7A11 and GPX4 expression compared with those in the Dex + I/R group. Interestingly, the expression of ferritin, TFR1, SLC7A11, GPX4, and ASCL4 in the myocardial tissue of the I/R group resembled that of the Dex + Yoh + I/R group (Fig. 4A–F).

### 3.5 Dex preconditioning enhanced antioxidant and reduced inflammation in rat myocardial tissues

**Fig. 5:**
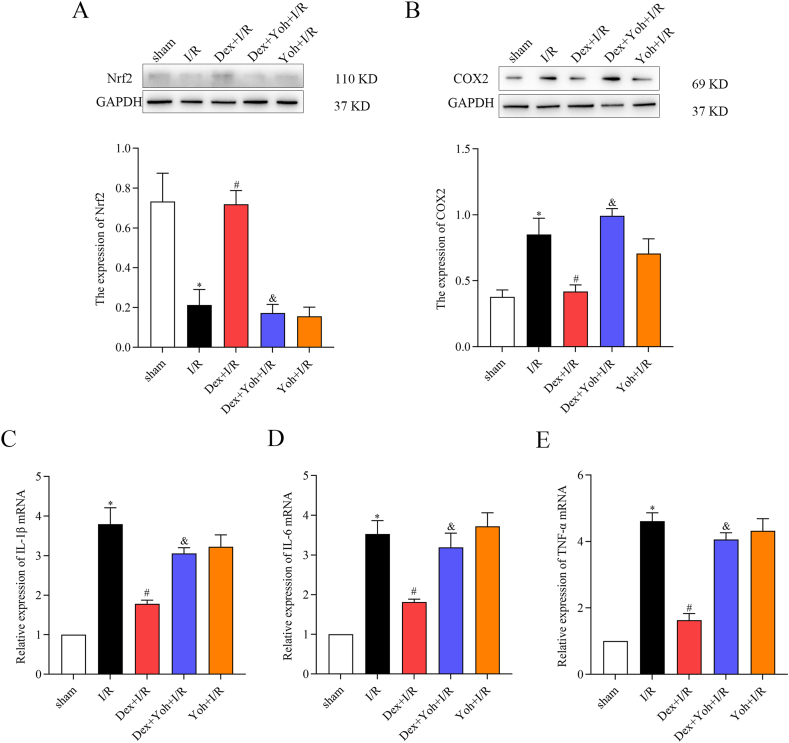
Dex preconditioning enhanced antioxidant activity and reduced inflammation in rat myocardial tissues. (A) Nrf2 protein level. (B) COX2 protein level. (C) IL-1β mRNA expression. (D) IL-6 mRNA expression. (E) TNF-α mRNA expression. n = 3/group. All data are presented as the mean ± SEM. ∗*P* < 0.05 *vs.* the Sham group. ^#^*P* < 0.05 *vs.* the I/R group. ^&^*P* < 0.05 *vs.* the Dex + I/R group.

Furthermore, Dex preconditioning demonstrated its ability enhanced antioxidant defenses and reduced inflammation in the rat myocardial tissues. Compared to the sham group, the I/R group showed decreased expression of Nrf2, along with increased levels of COX2 and mRNA levels of IL-1β, IL-6, and TNF-α. Intriguingly, Dex preconditioning upregulated Nrf2 expression, downregulated COX2 expression, and reduced mRNA levels of IL-1β, IL-6, and TNF-α. Notably, pretreatment with Yoh and Dex diminished these differences. Notably, the Yoh + I/R group exhibited similar protein and gene expression patterns to those observed in the Dex + Yoh + I/R group (Fig. 5A–E).

## 4 Discussion

In this study, we investigated whether Dex preconditioning can prevent MIRI by attenuating ferroptosis, and whether this effect depends on α2 adrenergic receptors in rats. Our results demonstrated that Dex preconditioning effectively improved cardiac function, reduced infarct size and pathological damage, and suppressed ferroptosis and inflammation in rat myocardium after I/R injury. These effects were associated with modulation of the Nrf2 and SLC7A11/GPX4 pathways. This study also highlighted the role of α2 adrenergic receptors in mediating the cardioprotective effects of Dex preconditioning against MIRI, as Yoh abolished the protective effects of Dex preconditioning.

MIRI, a critical factor influencing the prognosis of patients with myocardial infarction, underscores the importance of identifying approaches to alleviate I/R injury in the treatment of ischemic cardiovascular diseases. This study demonstrated that Dex significantly protected against MIRI in rats. It improved LVEF and LVFS, reduced myocardial infarct size, and lowered serum CK-MB levels. Dex preserved myocardial and mitochondrial integrity, mitigating MIRI-induced pathological changes. These benefits were nullified by Yoh, indicating that Dex's effects are mediated via α2 adrenergic activation. Thus, Dex offers notable cardioprotection in MIRI, with its efficacy strongly tied to α2 adrenergic pathways, highlighting its therapeutic potential for MIRI.

Recent research has shed light on the role of ferroptosis, which is regulated by the balance between lipid peroxidation and antioxidant defense in the pathogenesis of MIRI. Inhibition of ferroptosis has emerged as a promising strategy for cardioprotection [21,22]. Our study confirms previous findings that Dex can protect against MIRI by suppressing ferroptosis [17,18]. However, these studies primarily focused on Dex post-conditioning and did not explore the involvement of α2 adrenergic receptors in this process. To address this gap, we employed Dex preconditioning as a clinically relevant intervention and demonstrated its ability to prevent MIRI by inhibiting ferroptosis in rats. Importantly, we found that the protective effects of Dex preconditioning were mediated by α2 adrenergic receptors, as evidenced by the blockade of the beneficial effects of Dex by yohimbine.

SLC7A11 and GPX4 are crucial regulators of ferroptosis. SLC7A11 functions as a cystine/glutamate antiporter, facilitating the uptake of cystine, an amino acid required for GSH synthesis [23]. The reduction in SLC7A11 can lead to decreased cystine uptake and increased susceptibility to ferroptosis [24]. Conversely, GPX4 is a GSH-dependent enzyme that can eliminate lipid hydroperoxides, which are toxic byproducts of lipid peroxidation that can trigger ferroptosis [22]. Inhibition of GPX4 results in lipid peroxidation, leading to ferroptotic cell death during MIRI [25,26]. Furthermore, Yu et al. [18] showed that Dex post-conditioning inhibited MIRI-induced ferroptosis via SLC7A11/GPX4 activation in mice. We observed decreased levels of SLC7A11 and GPX4 in the myocardial tissue of the I/R group, indicating induction of ferroptosis in myocardial cells during I/R injury. Conversely, Dex preconditioning significantly activated SLC7A11/GPX4, demonstrating its potent antiferroptotic effects. However, when Yoh and Dex preconditioning were combined, the expression of SLC7A11 and GPX4 was lower than in the Dex + I/R group. This finding suggests that the anti-ferroptotic effect of Dex preconditioning is mediated through regulation of the SLC7A11/GPX4 axis via α2 adrenergic receptors.

ACSL4 is another important regulator of ferroptosis, and acts as an enzyme involved in polyunsaturated fatty acid synthesis related to lipid peroxidation [27]. Studies conducted in breast cancer cells have demonstrated that silencing ACSL4 can significantly reduce the production of polyunsaturated fatty acids and suppress ferroptosis [28]. In our study, we observed an upregulation of ACSL4 protein expression in the I/R group, indicating the induction of ferroptosis in myocardial cells during I/R injury. Remarkably, Dex preconditioning significantly decreased ACSL4 expression, highlighting its potent antiferroptotic effect. However, when Yoh and Dex preconditioning were combined, there was an increase in ACSL4 protein expression compared with that in the Dex + I/R group. This suggests that the anti-ferroptotic effect of Dex preconditioning is mediated by the regulation of ACSL4 via α2 adrenergic receptors.

Maintenance of iron homeostasis is critical for cardiac function. Excessive iron accumulation through the Fenton reaction during MIRI can induce ferroptosis by increasing ROS and lipid peroxidation [21]. Therefore, the transport, storage, and utilization of iron can influence cellular susceptibility to ferroptosis. Ferritin and TFR1 are critical proteins involved in these processes, and their expression levels have been shown to be affected during MIRI [29]. TFR1, an iron uptake protein that transports transferrin-bound iron into the cells, is vital for maintaining iron homeostasis. Knockdown of TFR1 suppresses erastin-induced ferroptosis, whereas upregulation of TFR1 can enhance iron uptake, potentially leading to iron overload [30]. TFR1 also maintains cardiomyocyte homeostasis, as animals with mutant TFR1 develop lethal dilated cardiomyopathy after birth [31]. In the present study, we observed iron accumulation and upregulated TFR1 expression in I/R rats, indicating ferroptosis activation following MIRI [[32], [33], [34]]. Dex preconditioning can attenuate these adverse effects by reducing intracellular iron levels, making it a potential therapeutic strategy for treating ferroptosis-induced cardiac damage.

Excessive iron accumulation in MIRI promotes lipid peroxidation and inflammation. Nrf2 is a transcription factor that protects cells against oxidative stress and regulates various proteins involved in ferroptosis, including GPX4, SLC7A11, and TFR1 [35,36]. Studies have demonstrated that Nrf2 can decrease the expression of SLC7A11, leading to the induction of ferroptosis [35]. Conversely, Nrf2 activation can inhibit ferroptosis [37,38]. Our findings suggest that Dex preconditioning decreases the levels of pro-oxidant molecules (MDA) and enhances antioxidant enzymes (Nrf2, SOD, and GSH) in the rat myocardium during I/R injury. This finding aligns with a study conducted by Wang et al. [17], who observed that Dex post-conditioning reduced MIRI-induced ferroptosis through the activation of Nrf2.

Inflammatory mediators, including COX2 and pro-inflammatory cytokines, are major contributors to MIRI as they can induce cell death and tissue damage. Dex preconditioning inhibited I/R-induced inflammatory response, as evidenced by the reduced expression of COX2 and inflammatory factors in the rat myocardium. This anti-inflammatory effect is clinically relevant, because inflammation is a critical factor that exacerbates myocardial injury during I/R. Moreover, the anti-inflammatory effects of Dex preconditioning may contribute to its ability to attenuate ferroptosis, because inflammation can promote ferroptosis by inducing oxidative stress and disrupting iron homeostasis. Therefore, by reducing inflammation, Dex preconditioning may alleviate oxidative stress and iron dysregulation and ultimately inhibit ferroptosis. These findings hold significant clinical importance given the pivotal role of inflammation in exacerbating myocardial injury during I/R.

Interestingly, the combination of Dex and Yoh appeared to counteract these effects, resulting in an increase in COX2 and pro-inflammatory cytokine levels. In the Yoh + I/R group, the absence of Dex means there is no activation of α2 adrenergic receptors, leading to elevated oxidative stress markers similar to those in the Dex + Yoh + I/R group. The comparable outcomes in these groups further corroborate the role of α2 adrenergic receptors in modulating these molecular pathways. Taken together, these findings offer valuable insights into the molecular mechanisms underlying the protective effects of Dex and its interaction with α2 adrenergic receptors in MIRI, emphasizing its potential as a therapeutic target for mitigating myocardial damage.

This study innovatively explores the effects of in vivo Dex preconditioning on MIRI, highlighting the role of the α2 adrenergic receptor and ferroptosis. Unlike established post-conditioning research, the use of yohimbine confirms that Dex's preconditioning benefits, such as enhanced cardiac function and reduced ferroptosis, depend on the α2 receptor. Additionally, it provides a detailed molecular analysis of ferroptosis-related markers (Nrf2/SLC7A11/GPX4) and inflammatory indicators, offering new insights into Dex-mediated cardioprotection. This comprehensive study significantly enhances the understanding of Dex's potential in preventing MIRI.

This study has several limitations that should be acknowledged. First, the use of a rat model of MIRI may not fully reflect the clinical scenario of MIRI in humans. Second, the α2 adrenergic receptor antagonist used in this study, yohimbine, may have some off-target effects. Third, the levels of ROS and iron in the myocardium were not measured directly but were inferred from the expression of related proteins. Lastly, the detailed molecular mechanisms by which Dex preconditioning activates the Nrf2 and SLC7A11 pathways via α2 adrenergic receptors were not explored in this study.

## 5 Conclusion

The present study demonstrated that Dex preconditioning confers cardioprotection against MIRI by attenuating ferroptosis and the inflammatory response through the activation of the Nrf2/ACSL4 and SLC7A11/GPX4 pathways via α2 adrenergic receptor activation. These observations imply that Dex preconditioning is a promising prospective therapeutic approach for MIRI in the clinical setting. Nonetheless, additional studies are necessary to elucidate the intricate molecular mechanisms underlying the activation of the Nrf2/ACSL4 and SLC7A11/GPX4 pathways by Dex preconditioning and to validate these findings in other models and species.

## CRediT authorship contribution statement

**Mingling Wang:** Writing – original draft, Methodology, Data curation, Conceptualization. **Zhuoyu Ren:** Writing – original draft, Methodology, Data curation, Conceptualization. **Xiaotong Sun:** Supervision, Methodology, Data curation, Conceptualization. **Yaozu Li:** Supervision, Methodology, Data curation, Conceptualization. **Zuolei Chen:** Writing – review & editing, Supervision, Project administration, Conceptualization.

## Data availability statement

Data will be made available on request.

## Funding

This study was supported by grants from the 10.13039/501100007129Natural Science Foundation of Shandong Province (ZR2020QH010) and Science and Technology Development Plan of Weifang (2020YX088).

## Declaration of competing interest

The authors declare that they have no known competing financial interests or personal relationships that could have appeared to influence the work reported in this paper.
