# Supplementary material for: Dexmedetomidine preconditioning attenuates ferroptosis in myocardial ischemia-reperfusion injury via α2 adrenergic receptor activation
Source: Heliyon. 2024 Oct 22;10(21):e39697. doi: 10.1016/j.heliyon.2024.e39697 (PMC11544042; doi:10.1016/j.heliyon.2024.e39697)
Supplement: Multimedia component 1 [file mmc1.docx]

**Original Image for Figure 4A**







Ferritin

17KD







34KD

GAPDH

**Original Image for Figure 4B**





95KD

TFR1





34KD

GAPDH

**Original Image for Figure 4C**








34KD

ASCL4

72KD

GAPDH

**Original Image for Figure 4D**

GPX4





10KD





GAPDH

34KD

**Original Image for Figure 4E**







55KD

SLC7A11

GAPDH

34KD

Original Image for Figure 5A







95KD

GAPDH

Nrf2

34KD

Original Image for Figure 5B





72KD

COX2





GAPDH

34KD
